# What Will Trained Nurses Gain by Joining the British Nurses' Association?

**Published:** 1889-07-13

**Authors:** 


### WHAT WILL TRAINED NURSES GAIN BY JOINING THE BRITISH NURSES' ASSOCIATION?

THIS question has been satisfactorily answered by Miss Lückes in an admirable brochure which everyone should read, as it deals in detail with all the reasons advanced by the British Nurses' Association to induce nurses to join its ranks. She believes that the nursing profession will lose a great deal more than it can possibly gain by affiliating itself with an unaccredited body outside the jurisdiction of the hospitals, and combats successfully the attempt to loosen her relations with her training school, of registering her vocation with that of the trained midwife, and of reducing competent and, possibly, incompetent nurses to a dead level of uniformity.

It is hardly to be supposed that in the face of the strong opposition that may be anticipated in the event of the British Nurses' Association applying for a Royal charter, that such legal authority to legislate for nurses will be conceded to it, but we have heard so much of the efforts of the society in this direction that it is refreshing to hear the other side of the question, especially as the opinions are concurred in by most leading authorities.

Miss Lückes considers that if the schemes proposed by the British Nurses' Association were successful a serious blow would be dealt at that steady progress which has marked the position of nurses in recent years, and render it increasingly difficult to distinguish between first and second rate qualifications, and that the minor objects of convalescent homes and register offices for nurses in want of employment are not needed because they are already met by existing institutions. She does not look upon uniformity of theoretical knowledge as a test of a nurse's capacity or training, as learned women are often the worst nurses, nor, indeed, are the authorities of the best training schools agreed or likely to agree as to the required standard. As every nurse has an apprenticeship of two or three years to serve, during which she has time to correct false impressions and to surmount failures, the conclusion arrived at by the authorities of the training school as to her capabilities must be infinitely more valuable than a diploma granted by any self-constituted body. It should be an object also to encourage the feeling of friendliness in a nurse to the hospital in which she has been trained. There is no doubt a community of interest between all who nurse the sick, but it is well that all nurses should bear in mind, and to their credit they seldom forget it, that they are working for their hospital, their *alma mater*, that they are the custodians of its most cherished traditions, and that they are bound to maintain its good name and credit in whatever part of the world they may be placed Then, again, the friendly competition between different training schools tends to the greater development of the art itself, and ensures steady progress in general as well as in special nursing. About the difficulty of protecting the public from incompetent nurses, on which so much stress is laid, we deny the allegation. Nowadays, without any system of registration, anyone requiring a nurse can ascertain her personal qualifications by her certificate, and if this is not enough ample information regarding her antecedents may be obtained at the hospital in which she had been trained, or the private institution connected with it. If persons pass themselves off for trained nurses, the people who employ them are themselves to blame for not asking for written evidence of the truth of their statements, but neither medical men nor others have any difficulty in the matter, since skilled nurses are kept at private institutions connected with most of the large hospitals in London and throughout the country. These matters and many others are fully discussed in the paper before us, and Miss Lückes winds up her observations by advising nurses not to ignore their respective training schools, which have proved their best friends in the past, for an unsubstantial promise of advantage. The advantages nurses enjoy to-day are mostly the result of the unwearied labours of these authorities, and the improvements yet hoped for in many directions must depend chiefly upon their continued individual exertions.

A WORD ABOUT WHISKY.—Messrs. Ferguson, of Reading, have sent us samples of their old Scotch whisky. Chemical analysis is a favourite method of testing the value of wines and spirits. A method equally good, and, perhaps, better, is that which is adopted by lovers of "pudding." The proof of that universally esteemed comestible is undoubtedly in the eating. Similarly, with all deference to the analysts, we would submit that "The Test of the whisky is in the drinking." An experienced taster, not a tippler, will give a better decision as to the presence or absence of fusel oil, the fineness of the aroma, the maturity of the spirit, and other qualities than even the analyst himself. To three such experienced tasters, two of them Scotch, and all of them possessed of palates unspoilt by regular drinking, even in small quantities, we have submitted samples of the whisky sent us by Messrs. Ferguson. They all pronounced it "decidedly good, fine and mellow for toddy, and mild for use as a stimulant for the sick." What better testimony is needed? If to this practical proof the results of a chemical analysis be added, drinkers of whisky will have all the assurance they need that the Messrs. Ferguson can supply them with an excellent brand.

